# CircRANBP17 modulated KDM1A to regulate neuroblastoma progression by sponging miR-27b-3p

**DOI:** 10.1515/med-2023-0672

**Published:** 2023-03-13

**Authors:** Lijun Zhao, Junying Fan, Chunyang Zhang, Zhenjun Zhang, Jun Dong

**Affiliations:** Department of Neurosurgery, The Second Affiliated Hospital of Soochow University, Suzhou City, Jiangsu Province, 215000, China; Department of Nephrology, The First Affiliated Hospital of Baotou Medical College, Baotou, China; Department of Neurosurgery, The First Affiliated Hospital of Baotou Medical College, Baotou, China; Department of Neurosurgery, The Second Affiliated Hospital of Soochow University, No. 1055, Sanxiang Road, Gusu District, Suzhou City, Jiangsu Province, 215000, China

**Keywords:** circRANBP17, miR-27b-3p, KDM1A, neuroblastoma

## Abstract

Neuroblastoma (NB) is a common childhood cancer. Circular RNA RAN binding protein 17 (circRANBP17) has been identified to participate in diverse tumor progression. This study aims to explore the function and mechanism of circRANBP17 in NB. The levels of circRANBP17, miR-27b-3p and KDM1A in NB tissues and cells were measured by qRT-PCR. Mouse model assay was performed to investigate the effect of circRANBP17 knockdown on tumor formation *in vivo*. The levels of circRANBP17 and KDM1A were significantly up-regulated, and the level of miR-27b-3p was strikingly down-regulated in NB tissues and cells (SK-N-SH and SK-N-AS). Functional studies indicated that miR-27b-3p inhibitor mitigated the inhibitory effects on cell proliferation, migration, invasion and the promoting effect on cell apoptosis in SK-N-SH and SK-N-AS cells induced by circRANBP17 knockdown. Also, miR-27b-3p regulated NB cell malignancy by targeting KDM1A. Further studies revealed that miR-27b-3p inhibitor reversed the low expression of KDM1A induced by circRANBP17 knockdown. In support, circRANBP17 knockdown led to inhibition of tumor formation *in vivo*. In conclusion, circRANBP17 modulated KDM1A to promote cell proliferation, migration, invasion and restrain cell apoptosis in NB by sponging miR-27b-3p, and the new regulatory network may provide a theoretical basis for the further study of NB.

## 1 Introduction

Neuroblastoma (NB) originates from the developing sympathetic nervous system, characterized by heterogeneous clinical behavior [1,2]. Despite the improvement of treatment strategies in recent years, the survival rate of high-risk NB remains 40–50% [3,4]. The main obstacle to NB treatment is high relapse and mortality [5]. Thus, it is necessary to further understand the mechanism behind NB progression.

Circular RNA (circRNA) is a conserved non-coding RNA and can function as an oncogene or tumor-repressing factor to regulate tumor progression [6]. Dysregulation of circRNAs is associated with cancers including NB. Zhang et al. revealed that circRNA cut like homeobox 1 (circCUX1) was strikingly up-regulated in NB tissues as well as cells, and its knockdown repressed tumor growth *in vivo* [7]. Fang and his colleagues reported that there were 29 dysregulated circRNAs in NB classes [8]. In another study, the malignant behaviors of NB cells were found to be related to circ_0132817/miR-432-5p/nucleolar protein 4 like (NOL4L) axis [9]. circRNA RAN binding protein 17 (circRANBP17) was an oncogene in nasopharyngeal carcinoma [10]. Moreover, we found that circRANBP17 was one of the top eight up-regulated circRNAs involved in the alteration in response to neuronal depolarization [11]. However, the mechanism of circRANBP17 was still unclear in NB.

MiRNAs, a class of non-coding RNAs, have been identified as tumor suppressors or carcinogens in many tumors including NB [12]. Zhu et al. indicated that miR-186-5p was down-regulated in NB patients, and its overexpression induced cell apoptosis and impeded cell proliferation; miR-186-5p overexpression inhibited tumor growth *in vivo* [13]. Another investigation about NB confirmed that miR-144-3p, weakly expressed in NB tissues, inhibited NB tumor progression *in vitro* [14]. The lysine-specific histone demethylase 1A (KDM1A, also named LSD1 or AOF2), a histone demethylase, is involved in many cancers [15,16]. Previous evidence confirmed that KDM1A promoted cell proliferation as well as motility in NB [17]. As a result, the biological mechanisms involving the function of miR-27b-3p and KDM1A in NB remain poorly understood.

Herein, we hypothesized that circRANBP17 induced KDM1A expression by interacting with miR-27b-3p to regulate NB cell phenotypes. We analyzed circRANBP17 expression in NB to determine its role in NB progression. The evidence from our study unraveled that circRANBP17 regulated KDM1A by targeting miR-27b-3p, so as to stimulate NB processes through regulation of cell proliferation, metastasis and apoptosis, shedding light on the mechanism of NB.

## 2 Materials and methods

### 2.1 Tissue sample

With the approval of the Ethics Committee of The Second Affiliated Hospital of Soochow University, 35 pairs of NB tissues and corresponding adjacent normal tissues were collected from The Second Affiliated Hospital of Soochow University. All tissues were frozen at −80°C. Informed consent was provided by NB patients or their guardians.

### 2.2 Cell culture and transfection

NB cell-lines SK-N-AS (CRL-2137), SK-N-SH (HTB-11), BE(2)-C (CRL-2268), IMR-32 (CCL-127) and human umbilical vein endothelial cell-line HUVEC (CRL-1730) were purchased from American Type Culture Collection (ATCC, Manassas, VA, USA). All cells were cultured in DMEM (Invitrogen, Carlsbad, USA) containing 10% fetal bovine serum (FBS, Thermo Fisher Scientific, Rockville, MD, USA) at 37°C in an incubator with 5% CO_2_. Small interference RNA (siRNA) targeting circRANBP17 (si-circRANBP17, 5′-GCCCAGTGTTTGCCAAACCTT-3′) and its mock (si-NC, 5′-CCTCTACCTGTCGCTGAGCTGTAAT-3′), miR-27b-3p mimics (miR-27b-3p, 5′-UUCACAGUGGCUAAGUUCUGC-3′) and its matched control (miR-NC, 5′-UUUGUACUACACAAAAGUACUG-3′), miR-27b-3p inhibitor (in-miR-27b-3p, 5′-GCAGAACUUAGCCACUGUGAA-3′) and its matched control (in-miR-NC, 5′-CAGUACUUUUGUGUAGUACAAA-3′) and KDM1A overexpression vector (KDM1A) and empty plasmid (pcDNA) were obtained from GenePharma (Shanghai, China). Lipofectamine 2000 Reagent (Invitrogen) was used for transfection process following the guidebook. The primers used to amplify the full length of coding sequence of KDM1A were as follows: 5′-GGAATTCCATGTTATCTGGGAAGAAGGC-3′ and 5′-CCGCTCGAGCGGTCACATGCTTGGGGACT-3′.

### 2.3 Quantitative real-time PCR (qRT-PCR)

One-step extraction method was utilized for isolation of total RNA with Trizol (Thermo Fisher Scientific). The specific steps were performed following the standard instructions. The concentration of RNA sample was detected using UV-3100PC spectrophotometer. Reverse transcription was conducted using cDNA synthesis kit (TaKaRa, Dalian, China). For the quantification of gene expression, SYBR Premix Ex Taq II (TaKaRa) was mixed with cDNA and reacted on qRT-PCR machine (Bio-Rad, Hercules, CA, USA) according to the standard methods. The relative expressions of circRANBP17, miR-27b-3p and KDM1A were calculated by the 2^−ΔΔCt^ method.

### 2.4 Western blot

The collected samples were lysed by Lysis Buffer (Thermo Fisher Scientific). After measure of protein concentrations with BCA Protein Assay Kit (Beyotime, Shanghai, China), an aliquot of 20 μg protein was loaded on bis-tris-acrylamide gels. The protein sample was transferred onto a PVDF membrane by using Bio-Rad blot apparatus. The membranes were incubated with primary antibodies and secondary antibodies (#31460/61-6520; 1:10,000; Thermo Fisher Scientific) in order, and exposed to a Hyperfilm ECL to develop protein blots. Abcam (Cambridge, MA, USA) provided all antibodies. The primary antibodies included anti-Bcl-2 (#MA5-11757; 1:50; Thermo Fisher Scientific), anti-Bax (#MA5-14003; 1:100; Thermo Fisher Scientific), anti-Cleaved-caspase 3 (#PA5-114687; 1:1,000; Thermo Fisher Scientific), anti-KDM1A (#PA5-17361; 1:1,000; Thermo Fisher Scientific), and anti-β-actin (#MA1-140; 1:8,000; Thermo Fisher Scientific).

### 2.5 Nucleocytoplasmic separation

NB cells were trypsinized and centrifuged at low speed. After washing with phosphate buffer solution, the cell pellet was placed on ice. Then, nucleocytoplasmic separation was performed with a PARIS™ Kit (Thermo Fisher Scientific) as instructed. The RNA from cytoplasm and nucleus was isolated and qRT-PCR was applied to quantify circRANBP17 expression. β-actin and U6 served as controls.

### 2.6 Identification of circRNA stability

The NB cells grown in 12-well plates were subjected to incubation with Actinomycin D (Amresco, Solon, OH, USA) for 0, 4, 8, 12, and 24 h. These cells were subsequently lysed and circRANBP17 consent was quantified by qRT-PCR. Linear RANBP17 acted as a reference.

### 2.7 Cell proliferation assay

For 3-(4,5-dimethyl-2-thiazolyl)-2,5-diphenyl-2-*H*-tetrazolium bromide (MTT) assay, the cells (5 × 10^3^ cells per well) were added into a 96-well plate and cultivated for 24, 48 and 72 h, followed by the incubation with MTT. Then, the cells were subjected to 10 min incubation with dimethyl sulfoxide. The optical density at 570 nm was read using a spectrophotometer (Thermo Fisher Scientific).

For 5-ethynyl-29-deoxyuridine (EdU) assay, SK-N-SH and IMR-32 cells were seeded in 12-well plates (1 × 10^5^ cells per well) and treated with test compounds. Meanwhile, 96-well plates were incubated with EdU-labeled medium for 2 h. After that, the cells were seeded into the 96-well plates, and the EdU-positive cells were analyzed by using cell proliferation detection kit (Ribobio, Guangzhou, China) following the guidebook.

For cell colony formation assay, the SK-N-SH and IMR-32 cells were grown in 6-well plates (500 cells per well). In brief, plasmids or oligonucleotides were transfected into the cells according to different aims. About 2-week culture later, the cells were incubated with paraformaldehyde (Phygene, Fuzhou, China) and crystal violet (Phygene), respectively. At last, the number of colonies greater than 1 mm in diameter was calculated to assess cell proliferation.

### 2.8 Wound-healing assay

NB cells were cultured in culture plates after various treatments. The cell wounds were created when cells were filled with the lower surface of the plates. Then, floated cells and debris were discarded, and the cells were cultured for 24 h. At last, the distance migrated by the cells was determined by analyzing the width of wounds under low-power microscope (Nikon, Tokyo, Japan).

### 2.9 Transwell assay

Transwell chambers were used to analyze cell migratory ability as per the method descripted by the manufacturer (Corning Tewksbury, MA, USA). In brief, NB cells were added into the upper chamber with serum-free DMEM. As chemoattractants, DMEM containing 10% FBS was placed into the lower chambers. After incubated for 24 h, the cells were fixed with 4% methanol, and then stained with 0.1% crystal violet. Cells were counted under a microplate (Nikon). The invasion assay was implemented based on the above methods except the cells were cultured in Matrigel chambers.

### 2.10 Cell apoptosis assay

After incubated for 24 h, the NB cells were digested with 0.25% trypsin. Then the fluorescence intensity of cells stained with Annexin V-FITC and propidium iodide were detected using Apoptosis Detection Kit (Solarbio, Beijing, China) to measure the apoptosis rate on a flow cytometry (Thermo Fisher Scientific).

### 2.11 Dual-luciferase reporter assay

The interactions between miR-27b-3p and circRANBP17 or KDM1A were predicted by starbase (http://starbase.sysu.edu.cn/agoClipRNA.php? source = mRNA) and DIANA tools (http://carolina.imis.athena-innovation.gr), respectively. The sequences of circRANBP17 and KDM1A 3′-UTR were amplified and then inserted into psiCHECK2 vector (Hanbio Biotechnology (Shanghai, China), named as circRANBP17 WT and KDM1A 3′-UTR WT, respectively. The miR-27b-3p mimics (miR-NC) and luciferase reporter (circRANBP17 WT, circRANBP17 MUT, KDM1A 3′-UTR WT or KDM1A 3′-UTR MUT) were co-transfected into SK-N-SH and IMR-32 cells. The luciferase activity was detected using Dual-Lucy Assay Kit (Solarbio).

### 2.12 RNA immunoprecipitation (RIP) assay

RIP assay was conducted using RIP assay Kit (Millipore, Bradford, MA, USA) according to the manual. In brief, the cell lysed fragment samples were incubated with magnetic beads conjugated with anti-Ago2 (Millipore) or anti-IgG (Millipore) antibody. Following that, the level of circRANBP17 was detected by qRT-PCR.

### 2.13 RNA pull-down assay

Biotinylated wild-type and mutant circRANBP17 (Bio-circRANBP17 WT and Bio-circRANBP17 MUT), and negative control (Bio-NC) were transfected into NB cells, respectively. After 48 h incubation, the cells were lysed using RIP lysis buffer, and incubated with streptavidin-coupled beads (Invitrogen) for 4 h. At last, qRT-PCR was used to determine miR-27b-3p expression.

### 2.14 Mouse model assay

The tumor xenograft investigation was performed with the approval of the Ethics Committee of The Second Affiliated Hospital of Soochow University. BALB/c nude mice (female; 5–6 weeks of age) were purchased from Charles River (Beijing, China), and arbitrarily divided into sh-NC and sh-circRANBP17 groups (*N* = 5 per group). Exclusion criteria were based on the differences of tumor volume and weight. The 5 × 10^6^ SK-N-SH cells stably expressing the small hairpin RNAs against circRANBP17 or negative control were adjusted to suitable concentration, and then subcutaneously injected into the right side of mice back. The volume of the forming tumors was measured every 1 week since the seventh day after injection as per volume (mm^3^) = width^2^ × length × 1/2. After 35 days, these mice were euthanatized, and the forming tumors were harvested for further analysis.

### 2.15 Immunohistochemistry (IHC) assay

The expression of KDM1A and nuclear proliferation marker (Ki-67) was evaluated in this part following the published methods [18]. Briefly, partial xenograft was sectioned to 4 μm thickness, and embedded into paraffin, followed by deparaffinization and hydration. Afterward, these slides were incubated with the primary antibody specific to KDM1A (#PA5-17361; 1:200; Thermo Fisher Scientific) or Ki-67 (#ab16667; 1:200; Abcam). Subsequent steps were conducted with an IHC assay kit (Phygene) following the guidebook. CX31-LV320 microscope (Olympus, Tokyo, Japan) was used to capture the staining results.

### 2.16 Statistical analysis

All data were analyzed using GraphPad Prism 7. Quantitative analysis was repeated for three times, and presented as mean ± standard deviation. Difference analysis in two groups was examined by Student’s *t*-test, and in more than three groups by one-way analysis of variance. Statistical significance is considered when *P* < 0.05.


**Ethics approval and consent to participate:** Written informed consents were obtained from all participants and this study was permitted by the Ethics Committee of The Second Affiliated Hospital of Soochow University.

## 3 Results

### 3.1 CircRANBP17 is significantly up-regulated in NB tissues and cells

**Figure 1:**
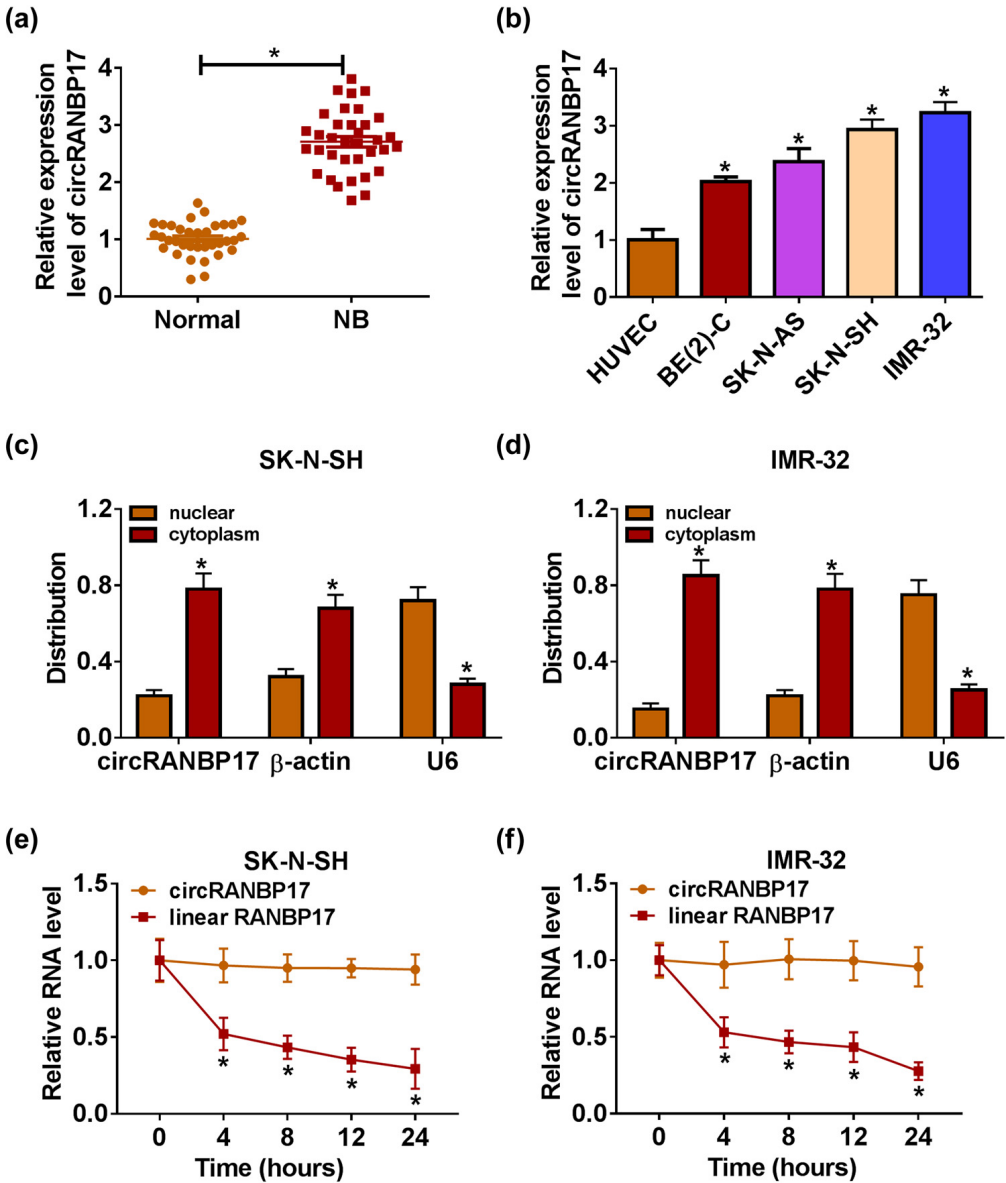
CircRANBP17 is significantly up-regulated in NB tissues and cells. The level of circRANBP17 in NB tissues (a) and cells (b) were detected by qRT-PCR. (c and d) Location of circRANBP17 in SK-N-SH and IMR-32 cells was confirmed by subcellular fractionation location assay. (e and f) Stability of circRANBP17 was identified by Actinomycin D assay. *P < 0.05.

To explore the role of circRANBP17 in NB, the relative expression of circRANBP17 was first measured by qRT-PCR. The results showed that the level of circRANBP17 was apparently increased in NB tissues in contrast to its expression in normal brain tissues (Figure 1a). Also, circRANBP17 was strikingly elevated in BE(2)-C, SK-N-AS, SK-N-SH and IMR-32 cells compared to that in HUVEC cells (Figure 1b). SK-N-SH and IMR-32 cells were employed for subsequent study as the higher expression of circRANBP17 in them. Additionally, we found that circRANBP17 was mainly located in cytoplasm of NB cells (Figure 1c and d). Furthermore, the data showed the high stability of circRANBP17 (Figure 1e and f). These results indicated that circRANBP17 was remarkably augmented in NB tissues and cells.

### 3.2 CircRANBP17 knockdown restrains cell proliferation, migration, invasion and induces cell apoptosis in SK-N-SH and IMR-32 cells

**Figure 2:**
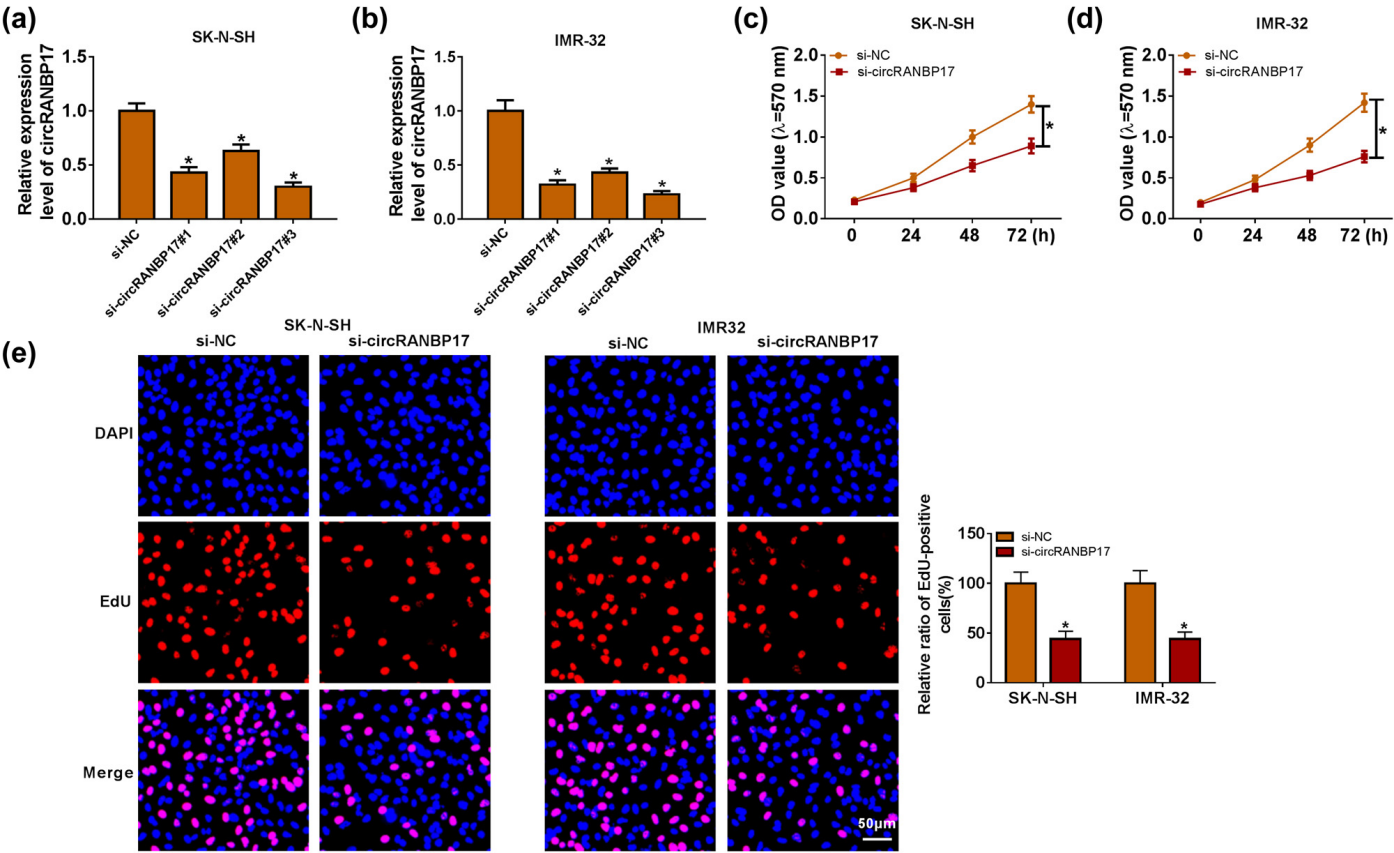
CircRANBP17 knockdown restrains cell proliferation in SK-N-SH and IMR-32 cells. The knockdown efficiency was detected by qRT-PCR in SK-N-SH (a) and IMR-32 (b) cells. The cell proliferative capacity (c–e) in SK-N-SH and IMR-32 cells transfected with si-circRANBP17 or si-NC were assessed by MTT and EdU assays. *P < 0.05.

**Figure 3:**
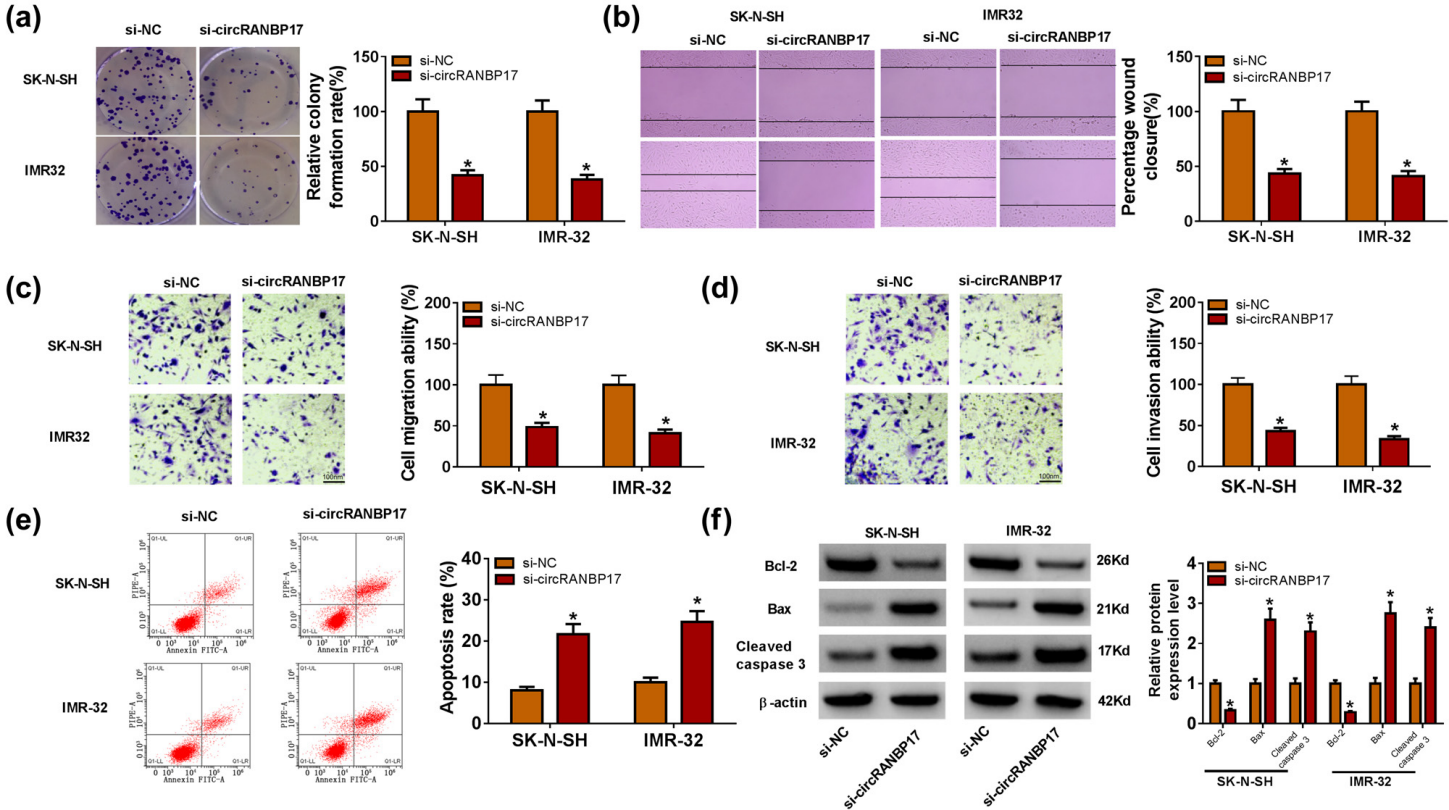
CircRANBP17 knockdown restrains cell migration and invasion and induces cell apoptosis in SK-N-SH and IMR-32 cells. The cell proliferative capacity (a), cell migration and invasion ability (b–d), and apoptosis rate (e) in SK-N-SH and IMR-32 cells transfected with si-circRANBP17 or si-NC were assessed by cell colony formation assays, wound-healing and Transwell assays and flow cytometry, respectively. The protein levels of Bcl-2, Bax and Cleaved caspase 3 were measured by western blot (f). *P < 0.05.

To illustrate the function of circRANBP17 in NB, circRANBP17 was silenced for the further exploration. The knockdown efficiency was confirmed by qRT-PCR, indicated by the evident down-regulation of circRANBP17 in SK-N-SH and IMR-32 cells (Figure 2a and b). Furthermore, the results exhibited that the cell proliferative capacity was inhibited after circRANBP17 knockdown (Figures 2c–e and 3a). Additionally, migration ability and invasion ability were all decreased in circRANBP17-knockdowned SK-N-SH and IMR-32 cells related to that in si-NC group (Figure 3b–d). Flow cytometry presented that the apoptosis rate was dramatically enhanced in SK-N-SH and IMR-32 cells transfected with si-circRANBP17 in comparison with that in si-NC group (Figure 3e). Since Bcl-2 acted as inhibitor and Bax, Cleaved caspase 3 functioned as proapoptotic factors in cell apoptosis [19], we investigated the effect of circRANBP17 silencing on the protein levels of Bcl-2, Bax and Cleaved caspase 3 by western blot assay. The results showed that the protein level of Bcl-2 was apparently decreased and the protein levels of Bax as well as Cleaved caspase 3 were notably increased in SK-N-SH and IMR-32 cells (Figure 3f). Taken together, the depletion of circRANBP17 inhibited cell proliferation, migration, invasion and promoted cell apoptosis in SK-N-SH and IMR-32 cells.

### 3.3 miR-27b-3p negatively interacts with circRANBP17

**Figure 4:**
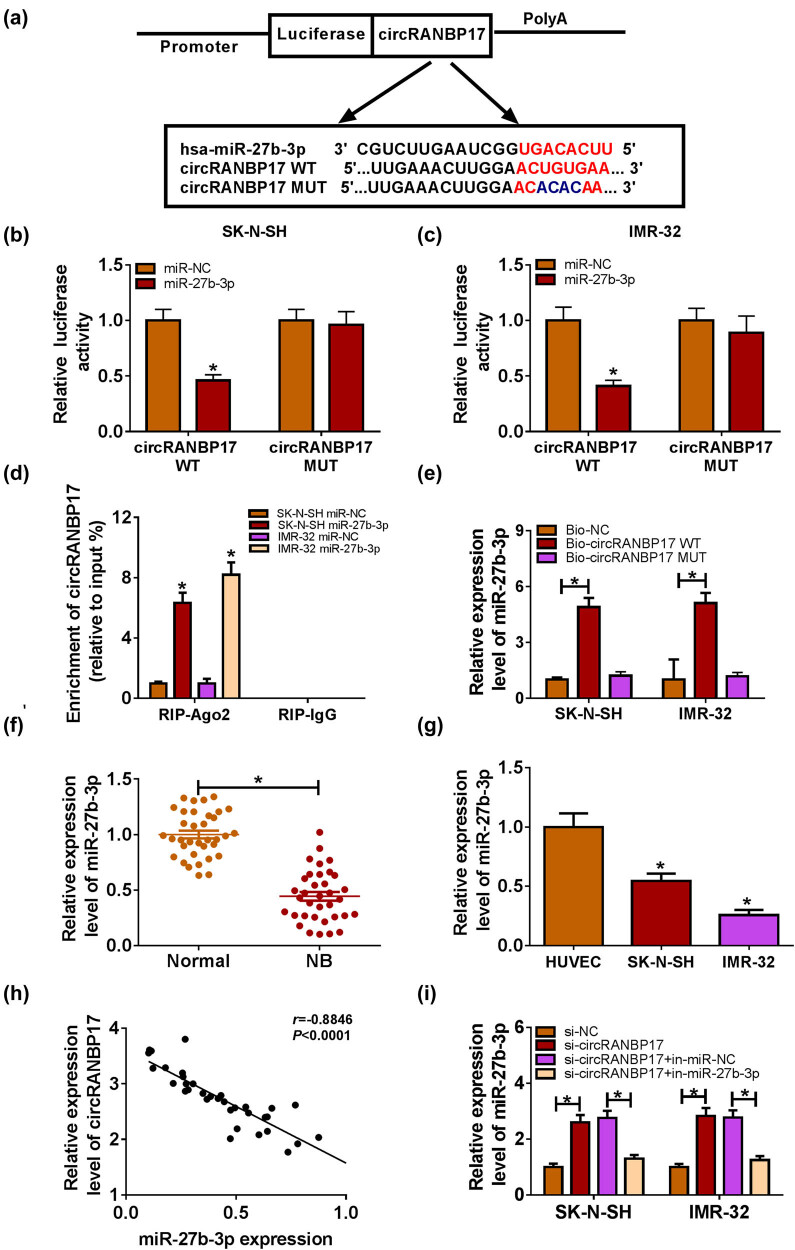
miR-27b-3p negatively interacts with circRANBP17. (a) Complementary binding sites between miR-27b-3p and circRANBP17. The interaction between miR-27b-3p and circRANBP17 was verified by dual luciferase reporter assay in SK-N-SH (b) and IMR-32 (c) cells. (d) Enrichment of circRANBP17 by Ago2 or IgG antibody was performed by RIP assay. (e) RNA pull-down assay was carried out to confirm the association of miR-27b-3p and circRANBP17. The level of miR-27b-3p in NB tissues (f) and cells (g) was measured by qRT-PCR. (h) Correlation between miR-27b-3p and circRANBP17. (i) Level of miR-27b-3p in both the SK-N-SH and IMR-32 cells transfected with si-NC, si-circRANBP17, si-circRANBP17 + in-miR-NC or si-circRANBP17 + in-miR-27b-3p. *P < 0.05.

**Figure A1:**
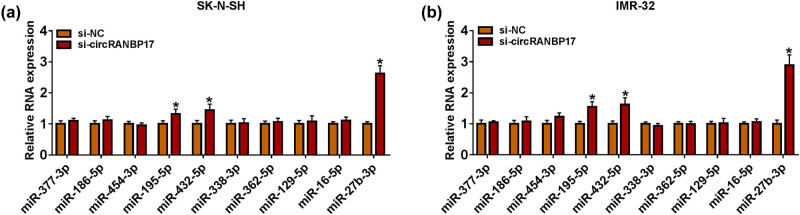
The miRNAs with circRANBP17-binding sites and related to NB progression were analyzed by qRT-PCR after circRANBP17 depletion in SK-N-SH (a) and IMR-32 cells (b). *P < 0.05.

To investigate the potential mechanism of circRANBP17 in NB, starbase tools online database was used to search the putative target of circRANBP17. The miRNAs with circRANBP17-binding sites and related to NB progression were analyzed by qRT-PCR after circRANBP17 depletion in SK-N-SH and IMR-32 cells. As shown in Figure A1a and b, circRANBP17 depletion significantly up-regulated miR-195-5p, miR-432-5p and miR-27b-3p, especially up-regulated miR-27b-3p. Thus, miR-27b-3p was chosen for the present study. The results showed that miR-27b-3p had complementary sequence of circRANBP17 (Figure 4a). Dual-luciferase reporter assay showed that the transfection of miR-27b-3p mimics resulted in the distinct reduction of luciferase activity of circRANBP17 WT, while luciferase activity had no apparent change in miR-NC group; however, the luciferase activity of circRANBP17 MUT reporter had no significant fluctuation in SK-N-SH and IMR-32 cells transfected with miR-27b-3p or miR-NC (Figure 4b and c). Moreover, the RIP assay indicated that circRANBP17 was notably enriched by Ago2 antibody in comparison with that in IgG control group (Figure 4d). RNA pull-down assay displayed that miR-27-3p was enriched in Bio-circRANBP17 WT group as compared with its expression in Bio-circRANBP17 MUT group (Figure 4e). In addition, the level of miR-27b-3p was evidently decreased in NB tissues and cells compared to that in their matched controls (Figure 4f and g). After analyzing the relationship between miR-27b-3p expression and clinicopathologic features of NB patients, we found that miR-27b-3p expression was associated with tumor-node-metastasis grade (Table A1). The scatter diagram showed that the level of circRANBP17 was negatively correlated with miR-27b-3p (Figure 4h). Further data displayed that miR-27b-3p was obviously increased in SK-N-SH and IMR-32 cells with the transfection of si-circRANBP17, which was attenuated after miR-27b-3p depletion (Figure 4i). These data manifested that miR-27b-3p was a direct target of circRANBP17.

### 3.4 miR-27b-3p inhibitor restrains the inhibitory effects on cell proliferation, migration, invasion and the promotion effect on cell apoptosis in SK-N-SH and IMR-32 cells induced by circRANBP17 knockdown

**Figure 5:**
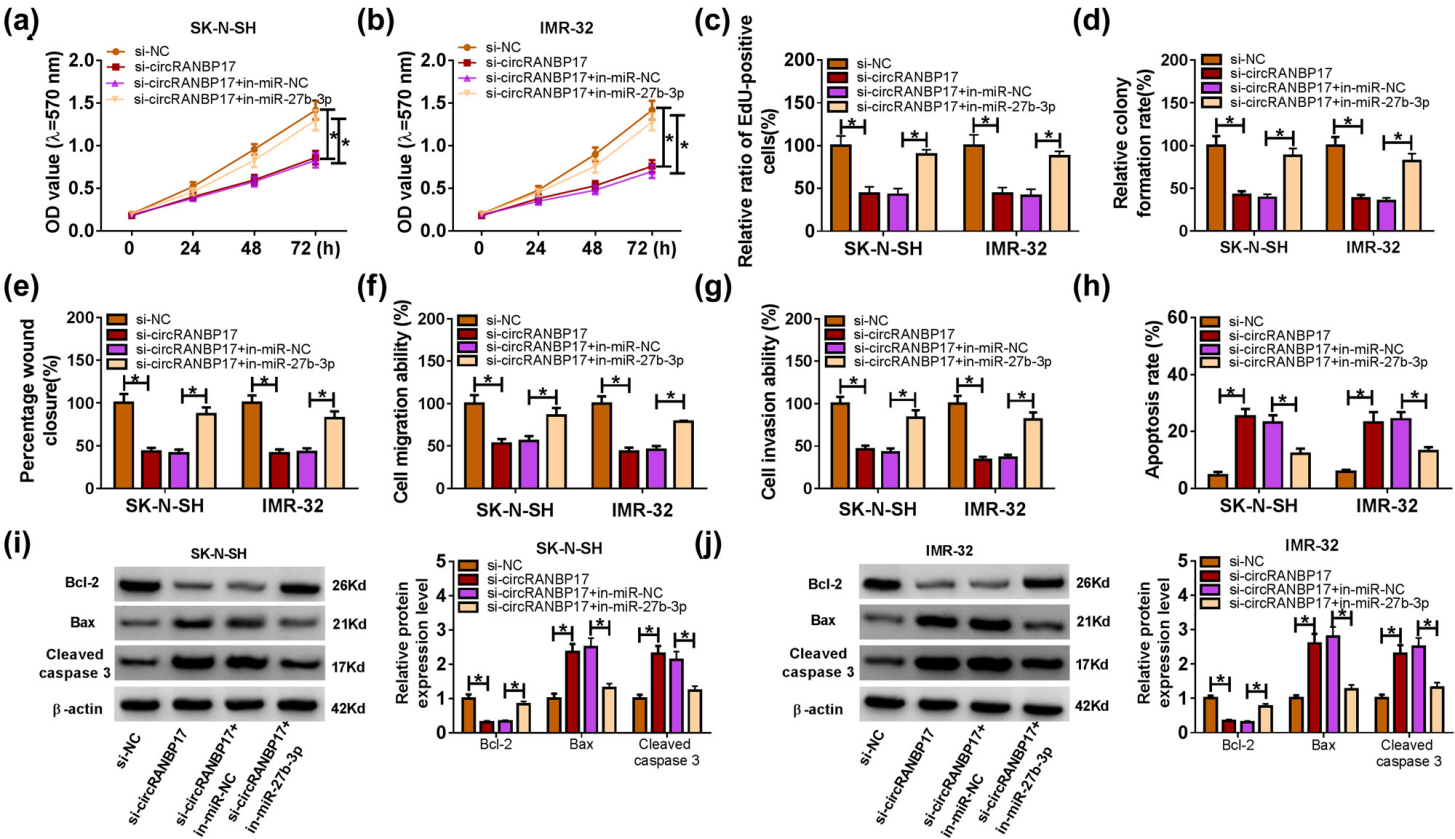
miR-27b-3p inhibitor restrains the inhibitory effects on cell proliferation, migration, invasion and the promoting effect on cell apoptosis induced by circRANBP17 knockdown in SK-N-SH and IMR-32 cells. Cell proliferative capacity (a–d), migration ability (e and f), invasion ability (g), apoptosis rate (h) and the protein levels of Bcl-2, Bax and Cleaved caspase 3 (i and j) in SK-N-SH and IMR-32 cells transfected with si-circRANBP17, si-circRANBP17 + in-miR-27b-3p, or their matched controls were assessed by MTT, EdU and cell colony formation assays, wound-healing assay, Transwell assay, flow cytometry and western blot, respectively. *P < 0.05.

To confirm the function of circRANBP17 and miR-27p-3p in NB, si-circRANBP17 and in-miR-27b-3p were co-transfected into SK-N-SH and IMR-32 cells. The data presented that the cell proliferative capacity was inhibited in circRANBP17-knockdown SK-N-SH and IMR-32 cells, which was rescued after miR-27b-3p absence (Figure 5a–d). Additionally, migration and invasion ability were all obviously down-regulated in si-circRANBP17-transfected SK-N-SH and IMR-32 cells, while the emergence of in-miR-27b-3p reversed the inhibitory effect (Figure 5e–g). However, the promoting effect of circRANBP17 absence on the apoptosis of SK-N-SH and IMR-32 cells was remitted by reduced expression of miR-27b-3p (Figure 5h–j). For instance, circRANBP17 absence led to the increase of apoptotic rate and the protein expression of Bax as well as Cleaved caspase 3, and the inhibition of Bcl-2 expression; however, these effects were relieved after miR-27b-3p depletion. These results revealed that miR-27b-3p inhibitor attenuated the inhibitory effects on cell proliferation, migration, invasion and the promotion effect on cell apoptosis in SK-N-SH and IMR-32 cells caused by circRANBP17 knockdown.

### 3.5 KDM1A negatively interacts with miR-27b-3p

**Figure 6:**
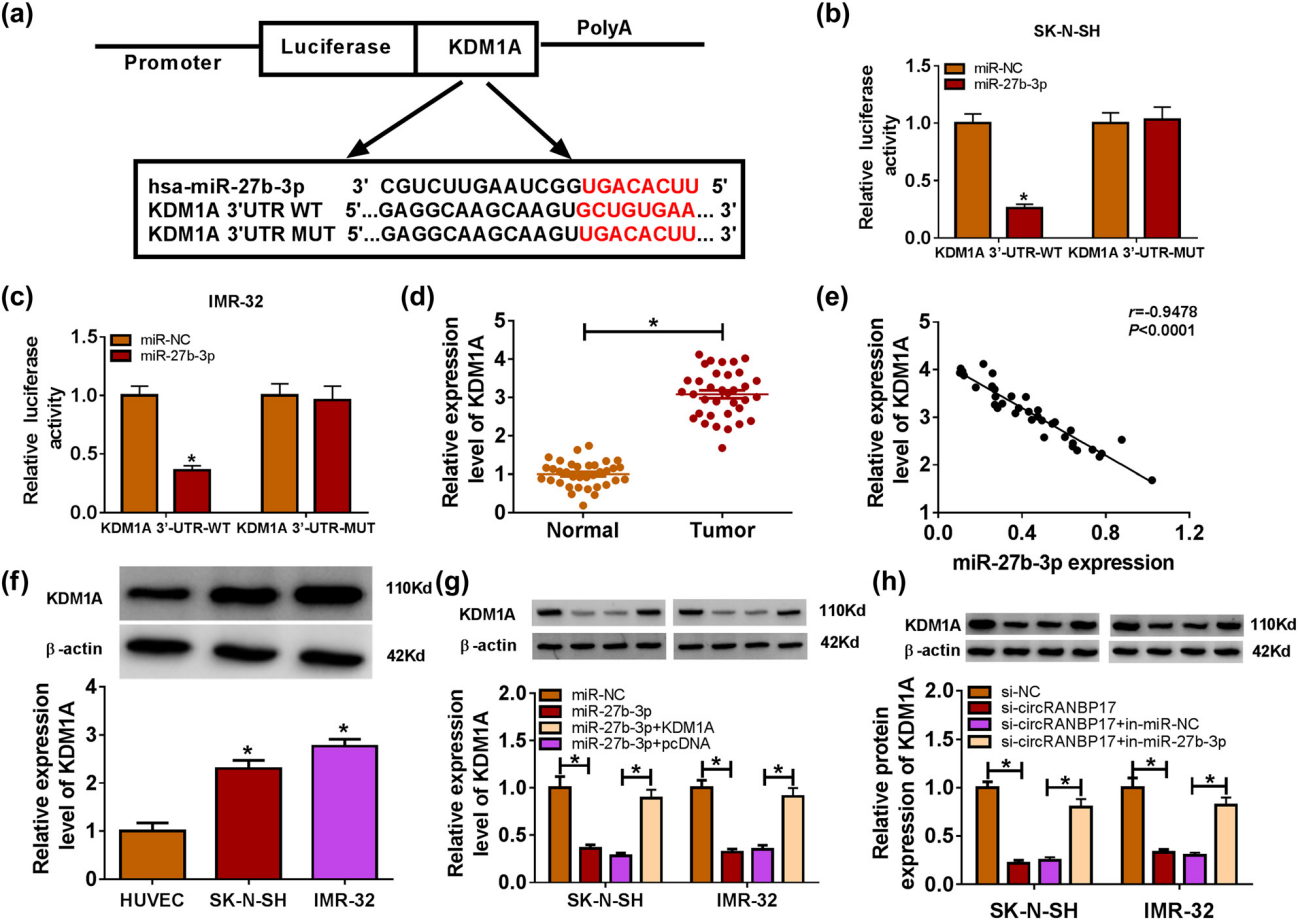
KDM1A negatively interacts with miR-27b-3p. (a) Complementary sequences between KDM1A 3′-UTR and miR-27b-3p. The luciferase activities of KDM1A 3′-UTR WT, KDM1A 3′-UTR MUT reporter in SK-N-SH (b) and IMR-32 (c) cells transfected with miR-27b-3p or miR-NC by dual luciferase reporter assay. The level of KDM1A in NB tissues (d) and cells (f) were detected by qRT-PCR. (e) Correlation between KDM1A and miR-27b-3p. (g) Protein level of KDM1A in SK-N-SH and IMR-32 cells after miR-27b-3p and KDM1A overexpression were measured by western blot assay. The protein level of KDM1A in SK-N-SH and IMR-32 cells transfected with si-circRANBP17, si-circRANBP17 + in-miR-27b-3p or their matched controls was detected by western blot (h). *P < 0.05.

To explore the underlying mechanism of miR-27b-3p in NB, DIANA tools online database was used to search the putative target of miR-27b-3p. The results exhibited that KDM1A 3′-UTR had complementary binding sites of miR-27b-3p (Figure 6a). The luciferase activity of KDM1A 3′-UTR-WT reporter was dramatically down-regulated in SK-N-SH and IMR-32 cells transfected with miR-27b-3p mimics, while the luciferase activity of KDM1A 3′-UTR-MUT reporter had no significant change (Figure 6b and c). In addition, the level of KDM1A was elevated in NB tissues and cells (Figure 6d and f). The scatter diagram indicated that KDM1A was negatively correlated with miR-27b-3p (Figure 6e). The protein level of KDM1A was drastically down-regulated in SK-N-SH and IMR-32 cells transfected with miR-27b-3p mimics, whereas the effect was restored after KDM1A overexpression (Figure 6g). Based on the above results, the relationship among circRANBP17, miR-27b-3p and KDM1A was further explored. The qRT-PCR and western blot assay results exhibited that KDM1A was effectively down-regulated in circRANBP17-knockdowned SK-N-SH and IMR-32 cells, while the level of KDM1A was reverted after transfection with in-miR-27b-3p (Figure 6h). These data unraveled that miR-27b-3p inhibitor reversed the low expression of KDM1A induced by circRANBP17 knockdown.

### 3.6 KDM1A overexpression mitigated the inhibitory effects on cell proliferation, migration, invasion and the promotion effect on cell apoptosis in SK-N-SH and IMR-32 cells caused by miR-27b-3p

**Figure 7:**
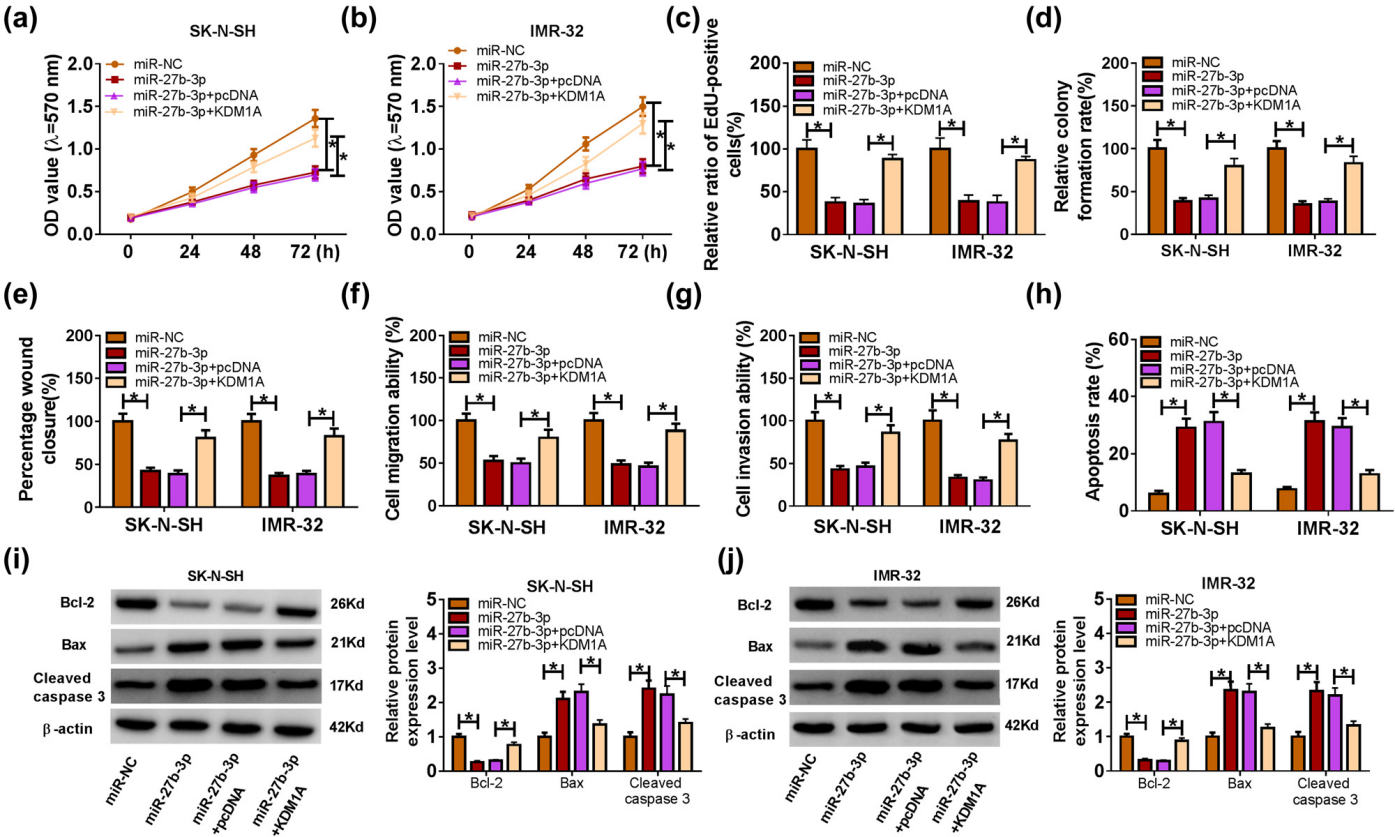
KDM1A overexpression mitigated the inhibitory effects on cell proliferation, migration, invasion and the promotion effect on cell apoptosis in SK-N-SH and IMR-32 cells by targeting miR-27b-3p. Cell proliferative capacity (a–d), migration ability (e and f), invasion ability (g), apoptosis rate (h) and the protein levels of Bcl-2, Bax and Cleaved caspase 3 (i and j) in SK-N-SH and IMR-32 cells transfected with miR-27b-3p, miR-27b-3p + KDM1A or their matched controls were assessed by MTT assay, EdU assay, cell colony formation assay, Transwell assay, flow cytometry and western blot, respectively. *P < 0.05.

To further explore the biological effect of miR-27b-3p and KDM1A in NB, miR-27b-3p and KDM1A were co-transfected into SK-N-SH and IMR-32 cells. Our data showed that cell proliferative capacity, migration ability and invasion ability were significantly decreased in SK-N-SH and IMR-32 cells, while the inhibitory effects were reversed by the emergence of KDM1A (Figure 7a–g). However, the cell apoptosis rate exhibited the opposite trend (Figure 7h). The western blot assay indicated that the protein level of Bcl-2 was strikingly decreased in SK-N-SH and IMR-32 cells transfected with miR-27b-3p mimics, while the level was regained in SK-N-SH and IMR-32 cells co-transfected with miR-27b-3p mimics and KDM1A; the protein levels of Bax, Cleaved caspase 3 showed the opposite trend (Figure 7i and j). These results implied that KDM1A overexpression alleviated the inhibitory effects on cell proliferation, migration, invasion and the promoting effect on cell apoptosis in SK-N-SH and IMR-32 cells induced by miR-27b-3p.

### 3.7 CircRANBP17 knockdown inhibited NB cell malignancy *in vivo*

**Figure 8:**
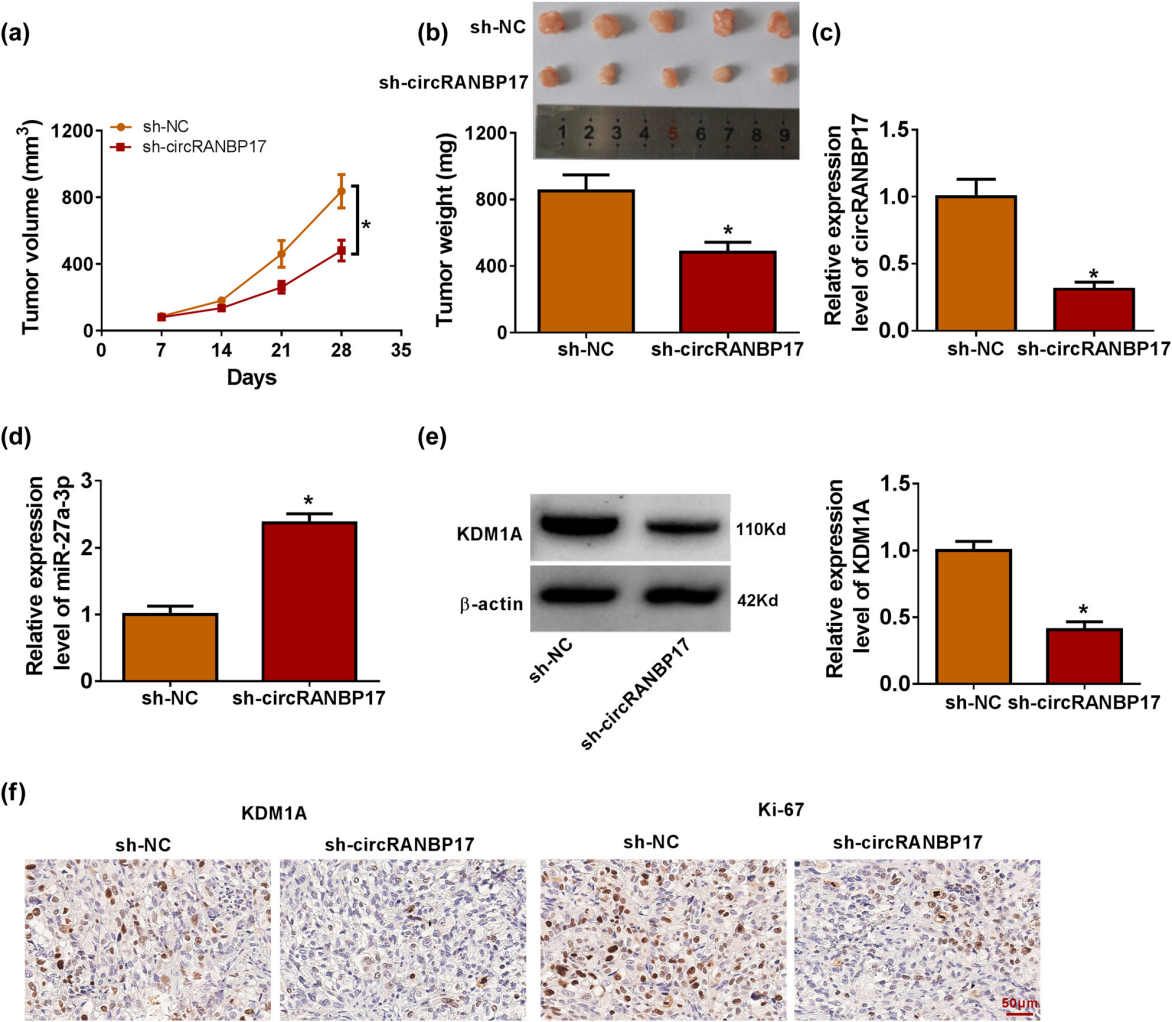
Effect of circRANBP17 knockdown on NB cell malignancy *in vivo*. (a and b) Effects of circRANBP17 depletion on tumor formation. (c–e) Impacts of circRANBP17 silencing on the expression of circRANBP17, miR-27b-3p and KDM1A were determined by qRT-PCR or western blot in the forming tumors from sh-circRANBP17 or sh-NC group. (f) Positive expression rates of KDM1A and Ki-67 were evaluated by IHC assay. *P < 0.05.

The *in vivo* assay was further performed to demonstrate the effect of circRANBP17 depletion on tumor formation. The data showed that reduced expression of circRANBP17 led to the delayed tumorigenesis (Figure 8a and b). Then, the study found that circRANBP17 and KDM1A expressions were down-regulated, while miR-27b-3p expression was up-regulated in the primary tumors from circRANBP17-knockdowned SK-N-SH cells (Figure 8c–e) as compared with controls. Also, the positive expression rate of both KDM1A and Ki-67 was lower in sh-circRANBP17 group than in sh-NC group (Figure 8f). These data demonstrated that circRANBP17 depletion inhibited NB cell malignancy *in vivo*.

## 4 Discussion

NB is a common malignant extracranial cancer in children. As reported, circRNAs play key roles in different types of tumors including NB. Based on the absent data about circRANBP17 in NB malignant progression, the study was organized to reveal circRANBP17’s role in NB and its underlying mechanism. As a result, we found that circRANBP17 could regulate NB progression via miR-27b-3p/KDM1A axis.

Accumulating evidence reported that NB malignant progression involved the altered expression of circRNAs, such as circ_0133622 [20], circRNA generated from AGO2 gene (circAGO2) [21] and circRNA TBC1 domain family member 4 (circTBC1D4) [22], which suggested the importance of circRNAs in NB progression. CircRANBP17, a novel circRNA, has been found to function as an oncogene in nasopharyngeal carcinoma [10]. In this work, we found circRANBP17 was located in cytoplasm of NB cells, and augmented in the clinical NB specimens and cells. Importantly, circRANBP17 silencing inhibited cell proliferative and migratory behaviors, and increased cell apoptotic rate. Besides, the altered levels of apoptosis-related Bcl-2, Bax and Cleaved caspase 3 also validated that the depletion of circRANBP17 promoted cell apoptosis in NB cells. Furthermore, *in vivo* assay also confirmed the repressing effect of circRANBP17 knockdown on tumor formation.

Previous study identified the dysregulation of miR-27b-3p in many diseases. For example, miR-27b-3p expression was decreased in oral lichen planus samples [23] and chronic liver injury [24]. miR-27b-3p was dramatically decreased in gastric cancer tissue as well as cell samples, and its introduction inhibited cell growth [25]. Herein, mechanism assay confirmed miR-27b-3p bound to circRANBP17. Following that, our data indicated the direct binding relationship of miR-27b-3p with circRANBP17. A comparative qRT-PCR analysis about NB tissues and cells confirmed miR-27b-3p was apparently decreased in clinical NB specimens and cell samples. miR-27b-3p expression was associated with tumor-node-metastasis grade. Meanwhile, it was found that circRANBP17 expression was negatively correlated with miR-27b-3p in clinical NB specimens. Moreover, the further functional study manifested that miR-27b-3p inhibitor could reverse circRANBP17-induced inhibition of cell proliferation and metastasis, and promotion of apoptosis.

KDM1A also reported to promote tumorigenesis in many cancers. For example, Pishas et al. explained KDM1A was obviously increased in Ewing sarcoma specimens and cells in comparison with controls [26]. In a study about prostate cancer, we found KDM1A was conspicuously increased in relapse prostate cancer tissues, and KDM1A silencing inhibited cell proliferation [27]. Actually, Yang et al. demonstrated KDM1A expression was distinctly enhanced in NB tissues, and KDM1A overexpression reversed the increased cell proliferation and metastasis caused by miR-329 [17]. Additionally, KDM1A combined with miR-542-3p to promote NB cell proliferation and invasion [28]. Here, we confirmed KDM1A was strikingly augmented in clinical NB specimens and cell samples. Mechanism assay validated KDM1A bound to miR-27b-3p. Meanwhile, KDM1A was negatively linear correlated with miR-27b-3p. Furthermore, the functional exploration indicated that KDM1A overexpression mitigated the decreased cell proliferative and metastatic capacities and increased apoptotic rate by miR-27b-3p in NB cells. In addition, *in vivo* data showed circRANBP17 knockdown repressed KDM1A production. Importantly, the mechanistic analysis revealed that circRANBP17 regulated KDM1A expression via absorbing miR-27b-3p in NB cells.

## 5 Conclusion

We found that circRANBP17 and KDM1A were remarkably up-increased, while miR-27b-3p was drastically decreased in clinical NB specimens and cell samples. Based on the all results, we found that circRANBP17 positively modulated KDM1A through binding to miR-27b-3p, thus regulating tumor progression in NB.

## Appendix

**Table A1:** Relationship between miR-27b-3p expression and clinicopathologic features of neuroblastoma patients

	Characteristics	miR-27b-3p expression		*P* value
	n=35	Low(n=17)	High(n=18)	
Age at diagnosis (years)				0.7332
≤3	14	6	8	
>3	21	11	10	
Sex				0.3175
Male	18	7	11	
Female	17	10	7	
TNM grade				0.0176*
I+II	16	4	12	
III+IV	19	13	6	

TNM, tumor-node-metastasis; **P* < 0.05.

## References

[1] Speleman F, Park JR, Henderson TO. Neuroblastoma: a tough nut to crack. Am Soc Clin Oncol Educ Book. 2016;35:e548–57. 10.1200/EDBK_159169 27249766

[2] Nakagawara A, Li Y, Izumi H, Muramori K, Inada H, Nishi M. Neuroblastoma. Jpn J Clin Oncol. 2018;48:214–41. 10.1093/jjco/hyx176 29378002

[3] Moreno L, Caron H, Geoerger B, Eggert A, Schleiermacher G, Brock P, et al. Accelerating drug development for neuroblastoma – new drug development strategy: an innovative therapies for children with cancer, European Network for Cancer Research in Children and Adolescents and International Society of Paediatric Oncology Europe Neuroblastoma project. Expert Opin Drug Discovery. 2017;12:801–11. 10.1080/17460441.2017.1340269 28604107

[4] Bagatell R, Cohn SL. Genetic discoveries and treatment advances in neuroblastoma. Curr Oppediatrics. 2016;28:19–25. 10.1097/MOP.0000000000000296 PMC4731097 26576010

[5] Fischer J, Pohl A, Volland R, Hero B, Dübbers M, Cernaianu G, et al. Complete surgical resection improves outcome in INRG high-risk patients with localized neuroblastoma older than 18 months. BMC Cancer. 2017;17:520. 10.1186/s12885-017-3493-0 PMC5543757 28778185

[6] Bach DH, Lee SK, Sood AK. Circular RNAs in cancer. Mol Ther Nucleic Acids. 2019;16:118–29. 10.1016/j.omtn.2019.02.005 PMC6411617 30861414

[7] Zhang X, Zhang J, Liu Q, Zhao Y, Zhang W, Yang H. Circ-CUX1 Accelerates the progression of neuroblastoma via miR-16-5p/DMRT2 Axis. Neurochem Res. 2020;45:2840–55. 10.1007/s11064-020-03132-w 33000435

[8] Zhang L, Zhou H, Li J, Wang X, Zhang X, Shi T, et al. Comprehensive characterization of circular RNAs in neuroblastoma cell lines. Technol Cancer Res Treat. 2020;19:1533033820957622. 10.1177/1533033820957622 PMC7533920 33000697

[9] Fang Y, Yao Y, Mao K, Zhong Y, Xu Y. Circ_0132817 facilitates cell proliferation, migration, invasion and glycolysis by regulating the miR-432-5p/NOL4L axis in neuroblastoma. Exp Brain Res. 2021;239:1841–52. 10.1007/s00221-021-06091-y 33837793

[10] Zhou M, Zhang P, Zhao Y, Liu R, Zhang Y. Overexpressed circRANBP17 acts as an oncogene to facilitate nasopharyngeal carcinoma via the miR-635/RUNX2 axis. J Cancer. 2021;12:4322–31. 10.7150/jca.55794 PMC8176428 34093832

[11] Mahmoudi E, Kiltschewskij D, Fitzsimmons C, Cairns MJ. Depolarization-associated CircRNA regulate neural gene expression and in some cases may function as templates for translation. Cells. 2019;9:25. 10.3390/cells9010025 PMC7017197 31861825

[12] Esteller M. Non-coding RNAs in human disease. Nat Rev Genet. 2011;12:861–74. 10.1038/nrg3074 22094949

[13] Zhu K, Su Y, Xu B, Wang Z, Sun H, Wang L, et al. MicroRNA-186-5p represses neuroblastoma cell growth via downregulation of Eg5. Am J Transl Res. 2019;11:2245–56. PMC6511750 31105832

[14] Cao XY, Sun ZY, Zhang LJ, Chen MK, Yuan B. microRNA-144-3p suppresses human neuroblastoma cell proliferation by targeting HOXA7. Eur Rev Med Pharmacol Sci. 2019;23:716–23. 10.26355/eurrev_201901_16885 30720179

[15] Shi Y, Lan F, Matson C, Mulligan P, Whetstine JR, Cole PA, et al. Histone demethylation mediated by the nuclear amine oxidase homolog LSD1. Cell. 2004;119:941–53. 10.1016/j.cell.2004.12.012 15620353

[16] Hayami S, Kelly JD, Cho HS, Yoshimatsu M, Unoki M, Tsunoda T, et al. Overexpression of LSD1 contributes to human carcinogenesis through chromatin regulation in various cancers. Int J Cancer. 2011;128:574–86. 10.1002/ijc.25349 20333681

[17] Yang H, Li Q, Zhao W, Yuan D, Zhao H, Zhou Y. miR-329 suppresses the growth and motility of neuroblastoma by targeting KDM1A. FEBS Lett. 2014;588:192–7. 10.1016/j.febslet.2013.11.036 24316513

[18] Huang DW, Huang M, Lin XS, Huang Q. CD155 expression and its correlation with clinicopathologic characteristics, angiogenesis, and prognosis in human cholangiocarcinoma. OncoTargets Ther. 2017;10:3817–25. 10.2147/OTT.S141476 PMC5546808 28814880

[19] Karch J, Molkentin JD. Regulated necrotic cell death: the passive aggressive side of Bax and Bak. Circ Res. 2015;116:1800–9. 10.1161/CIRCRESAHA.116.305421 PMC4443748 25999420

[20] Yang J, Yu L, Yan J, Xiao Y, Li W, Xiao J, et al. Circular RNA DGKB promotes the progression of neuroblastoma by targeting miR-873/GLI1 axis. Front Oncol. 2020;10:1104. 10.3389/fonc.2020.01104 PMC7390925 32793474

[21] Chen Y, Yang F, Fang E, Xiao W, Mei H, Li H, et al. Circular RNA circAGO2 drives cancer progression through facilitating HuR-repressed functions of AGO2-miRNA complexes. Cell Death Differ. 2019;26:1346–64. 10.1038/s41418-018-0220-6 PMC6748083 30341421

[22] Lin W, Wang Z, Wang J, Yan H, Han Q, Yao W, et al. circRNA-TBC1D4, circRNA-NAALAD2 and circRNA-TGFBR3: selected key circRNAs in neuroblastoma and their associations with clinical features. Cancer Manag Res. 2021;13:4271–81. 10.2147/CMAR.S297316 PMC8168971 34093041

[23] Chen J, Wang Y, Du G, Zhang W, Cao T, Shi L, et al. Down-regulation of miRNA-27b-3p suppresses keratinocytes apoptosis in oral lichen planus. J Cell Mol Med. 2019;23:4326–37. 10.1111/jcmm.14324 PMC6533518 30973209

[24] Li W, Chang N, Tian L, Yang J, Ji X, Xie J, et al. miR-27b-3p, miR-181a-1-3p, and miR-326-5p are involved in the inhibition of macrophage activation in chronic liver injury. J Mol Med (Berlin, Ger). 2017;95:1091–105. 10.1007/s00109-017-1570-0 28748390

[25] Tao J, Zhi X, Zhang X, Fu M, Huang H, Fan Y, et al. miR-27b-3p suppresses cell proliferation through targeting receptor tyrosine kinase like orphan receptor 1 in gastric cancer. J Exp Clin Cancer Res CR. 2015;34:139. 10.1186/s13046-015-0253-3 PMC4650850 26576539

[26] Pishas KI, Drenberg CD, Taslim C, Theisen ER, Johnson KM, Saund RS, et al. Therapeutic targeting of KDM1A/LSD1 in ewing sarcoma with SP-2509 engages the endoplasmic reticulum stress response. Mol Cancer Ther. 2018;17:1902–16. 10.1158/1535-7163.MCT-18-0373 PMC6201274 29997151

[27] Kashyap V, Ahmad S, Nilsson EM, Helczynski L, Kenna S, Persson JL, et al. The lysine specific demethylase-1 (LSD1/KDM1A) regulates VEGF-A expression in prostate cancer. Mol Oncol. 2013;7:555–66. 10.1016/j.molonc.2013.01.003 PMC3661758 23384557

[28] Wei Q, Guo Z, Chen D, Jia X. miR-542-3p suppresses neuroblastoma cell proliferation and invasion by downregulation of KDM1A and ZNF346. Open Life Sci. 2020;15:173–84. 10.1515/biol-2020-0018 PMC8114778 33987474

